# Impact of Interrupting Oral Prevention on Dental Health of 7- to 8-Year-Old Children Due to COVID-19

**DOI:** 10.3390/children12030315

**Published:** 2025-02-28

**Authors:** Julia Winter, Thea Hartmann, Constanze Schul, Esther Hörschgen, Miriam Thöne-Mühling, Birgit Wollenberg, Stefanie Amend, Roland Frankenberger

**Affiliations:** 1 Department of Operative Dentistry, Endodontics, and Pediatric Dentistry, Medical Center for Dentistry, University Medical Center Giessen and Marburg, Campus Marburg, Georg Voigt Str. 3, 35039 Marburg, Germany; hartmannthea@gmx.de (T.H.); frankbg@med.uni-marburg.de (R.F.); 2 Public Health Department in the District of Marburg Biedenkopf, Schwanallee 23, 35037 Marburg, Germany; schulc@marburg-biedenkopf.de (C.S.); hoerschgene@marburg-biedenkopf.de (E.H.); thoene-muehlingm@marburg-biedenkopf.de (M.T.-M.); wollenbergb@marburg-biedenkopf.de (B.W.); 3 Department of Pediatric Dentistry, Medical Center for Dentistry, University Medical Center Giessen and Marburg, Campus Giessen, Justus-Liebig-University Giessen, Schlangenzahl 14, 35392 Giessen, Germany; stefanie.amend@dentist.med.uni-giessen.de

**Keywords:** caries preventive program, schoolchildren, caries increment, COVID-19

## Abstract

**Background/Objectives**: In Marburg (Hesse, Germany), the selective intensive preventive program (SIP) with fluoride varnish applications had to be interrupted due to the COVID-19 pandemic. The aim of this retrospective anonymized evaluation was to investigate possible effects of SIP interruption on oral health in socially vulnerable 7- and 8-year-olds. **Methods**: The caries increment in 7- and 8-year-olds for the test group (N = 180) between last dental check-up before the interruption of SIP (02/2019–02/2020) and the first check-up after restart (01/2022–07/2022) were calculated from dental public health service data. The test group was compared to a control group of children (N = 215; same age and schools, with SIP, data collected between the school year 2017/18 and 2019/20). One dentist conducted the dental examinations. The University of Marburg ethics committee approved the study. The Mann–Whitney U test and Pearson’s chi-square test were used for statistical analysis. **Results**: There was no significant difference in the caries increment in the first dentition between the test and the control group for both age groups. In the different groups, a maximum of 61% of the children with caries experience were completely treated. There was no significant difference between the test and control groups in either the percentage of sealed first permanent molars or the degree of restoration. **Conclusions**: The interruption of SIP had no negative impact on caries increment. It is possible that the children examined went through the pandemic without a significant increase in tooth decay because the children were well-trained in tooth brushing since kindergarten.

## 1. Introduction

### 1.1. History of Group Prevention in the Study Setting

In the late 1970s, Prof. H.F.M. Schmidt (Department of Pediatric Dentistry at Marburg Dental School, Hesse, Germany) and his team developed a basic preventive program for schoolchildren that involved regular applications of the fluoride varnish Duraphat^®^ (Colgate-Palmolive GmbH, Hamburg, Germany). In the 1981/82 school year, this basic prophylaxis was offered for the first time to all children starting school in the city of Marburg and was extended to include the first classes in each of the following school years. In the 1992/93 school year, the basic preventive program was extended to the entire Marburg-Biedenkopf district. The effectiveness of this concept (“Marburg Model”) has been documented in various studies [1,2,3] and was recommended by the national associations of health insurance funds as a basic preventive program for group prevention in 2000.

The basic preventive program includes a school visit by the dental public health service per school half-year and a child-friendly presentation of a tooth-related educational topic, tooth brushing training in the class, and the fluoride varnish application of Duraphat^®^ (after parental consent). A dental examination is carried out once per school year.

Based on the examination data, it could be shown that not all schoolchildren benefit equally from the basic prophylaxis program [1]. Children with an increased caries risk, often living in socially deprived areas, had a lower caries reduction compared to children with a low caries risk. Since the 1995/96 school year, this unequal distribution of caries has been counteracted by a selective intensive preventive program (SIP) developed based on the Marburg Model. The SIP includes the usual dental examinations in the school setting, the presentation of dental topics in class (twice a year), the tooth brushing training under supervision in class (four times a year) and the fluoride varnish application (four times a year) [4,5]. The effectiveness of the intensive preventive program has been scientifically proven for fourth and sixth graders [5,6].

Since children starting school in socially deprived areas already had significantly higher dmf-t and DMF-T scores than children in other areas, an intensive preventive program has been offered in such areas since the 1999/2000 school year. This includes tooth brushing, nutrition units, dental examination, fluoride varnish applications, parental work and further training for kindergarten staff.

### 1.2. Changes Due to COVID-19

Due to the COVID-19 pandemic, the group prevention in kindergartens and schools located in socially deprived areas had to be interrupted. A German health insurance company (Kaufmännische Krankenkasse, KKH) reported a 12% decline in dental checkups for six- to twelve-year-olds from the first half of 2019 to the first half of 2020 [7]. In addition, studies reported on a change towards a negative behavior, such as eating and dental care behavior during the lockdown [8], which led to an increasing risk of caries [9]. There are so far only a few internationally published findings on the dental health status of children after COVID-19, and most studies only evaluate oral health habits [10]. In a Greek study, dental examination after the lockdown showed significantly increased mean dmf-t/DMF-T values in both the first and second dentition compared to the clinical examination of the same children 6 months before the lockdown [10]. An epidemiological study in Slovenia showed a lower dmf-t/DMF-T index after the pandemic compared to data from the school year 2018/2019 [11]. The authors of this paper did not find any papers in their literature search on PubMed that dealt with the actual increase in caries based on the individual child during the COVID-19 pandemic. Instead, the publications compared data on caries experience. Ideally, the caries increment in children between the last dental examination before the lockdown and the first examination after the restart of group prevention as the test group should be compared with the caries increment of a control group. Regarding the age and observation period for calculating caries increment, there should be no significant differences between the test and control groups, and calibrating the examiner would also be desirable. Due to the unforeseeable COVID-19 pandemic, prospective study planning was of course out of the question. It will hardly be possible to evaluate a data set that was collected continuously and contains the same observation period and the same age of the children for the test and control group. In the oral health data of the dental public health service in the district of Marburg-Biedenkopf, it may be possible to generate a test and a control group that meet or at least approximately meet the above criteria in the data collected by one examiner (T.H.) in socially deprived areas where selective intensive group prevention was performed. The aim of this retrospective anonymized data analysis was to investigate possible effects of SIP interruption on oral health of 7- and 8-year-olds in socially deprived areas. The null hypothesis was formulated as there being no difference in caries increment before the COVID-19 pandemic compared to during the pandemic.

## 2. Materials and Methods

**Figure 1:**
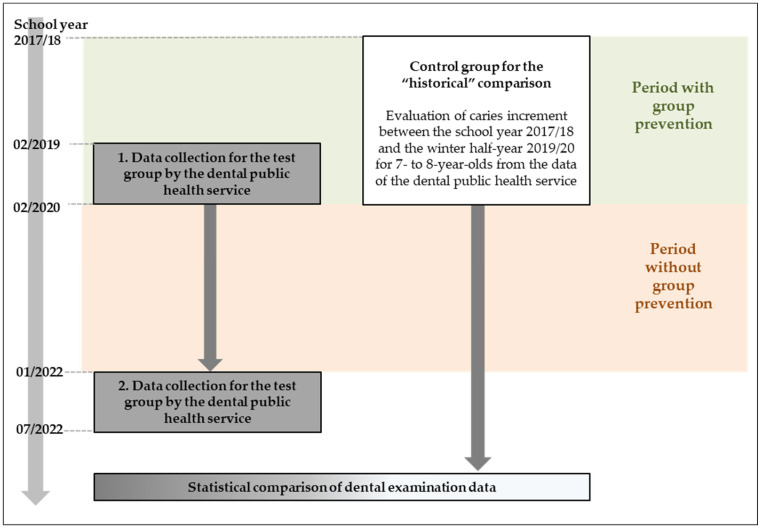
Flow chart of the retrospective anonymized data analysis of the data from the dental public health service on the effects of the COVID-19 pandemic on the dental health of 7- to 8-year-olds in socially deprived areas in the district of Marburg-Biedenkopf.

After approval by the local ethics committee (University of Marburg; file number 24-42-BO), a retrospective, anonymized data analysis was performed using data from the dental public health service. For this purpose, dental examination data from a test (N = 180) and a control group (N = 215) were compared regarding caries experience, caries increment, percentage of fissure sealings and level of oral rehabilitation. For the test group, anonymized oral examination data of 7- and 8-year-olds between the last screening before group prevention was interrupted (February 2019 to February 2020) and the restart of group prevention (January 2022 to July 2022) were used. For the control group, children of the same age from the same primary schools in socially deprived areas were included, whose data were collected between the 2017/18 school year and the winter of 2019/20 (Figure 1).

For this data analysis, data were included that were continuously collected during the period for the test and control groups by only one examiner (T.H.). The examiner (T.H.) was not specifically calibrated for this data analysis, but participated in all previous calibrations for the examinations in Hesse as part of the DAJ studies (Deutsche Arbeitsgemeinschaft Jugendzahnpflege e. V., German Working Group for Youth Dental Public Health) [12,13].

Before the interruption of SIP, the teeth were brushed with toothpaste prior to the dental examination. Due to the special hygiene regulations during the COVID-19 pandemic, teeth were not brushed after the restart of group prevention. The children were seated on a chair with their head leaning back. For the dental examination, plane mouth mirrors and a halogen spotlight (LEDental, PowerLight lite, Zossen, Germany, color temperature 5500 K, unit of illumination at 35 cm distance: 20,000 Lux) were used. The teeth were dried neither with cotton rolls nor with compressed air. The children were asked to swallow their saliva. For removing remaining biofilm and judging the quality of restoration margins, a blunt dental probe was used. The caries experience was recorded according to the World Health Organization (WHO) criteria using the dmf-t index [14]. Additionally, initial carious lesions, pit and fissure sealings, traumatized teeth and developmentally derived hypomineralization were noted. In the context of the screenings in the school setting, only visual caries diagnostics were performed, and no instrumental caries diagnostics such as fiber-optic transillumination or bitewing radiographs were used.

Due to legislation in the federal state of Hesse, no parental consent is required for the collection of dental oral health data by the dental public health service of the Marburg-Biedenkopf district [15], and anonymized data transfer to third parties for scientific data analysis is permitted [15,16]. In this context, a cooperation agreement was concluded between the dental public health service and the Department of Operative Dentistry, Endodontics and Pediatric Dentistry at Marburg Dental School. Among other things, this contract regulated the anonymization of the data by the staff of the dental public health service (C.S., E.H., M.T.-M.) and the transfer of the data for the data analysis (J.W.). Prior to anonymizing a data set consisting of two oral health assessments, the findings of the first and second examinations were checked by the staff of the dental public health service. In the case of the control group, findings queries could be checked against the available interim examinations and corrected in the event of clear data entry errors. The following extracted data were entered into Microsoft Office 365 Excel spreadsheets (Microsoft Corporation, Redmond, WA, USA): age of the child, dental examination on tooth level (dmf-t/DMF-t index), number of teeth with initial carious lesions including caries progression or arrest, number of first and second permanent molars with fissure sealants, caries risk measured by the criteria of the DAJ) [17] as a yes/no decision, degree of dental restorative rehabilitation, period of time between first and second dental examination, and caries increment between first and second dental examination (Δ dmf-t/Δ DMF-T).

A statistical evaluation was carried out with BioStat Pro (AnalystSoft Inc., BioStat Statistical Analysis Program version v7, 340 S Lemon Ave # 3010, Walnut, CA, USA). The Mann–Whitney U test and Pearson’s chi-square test were used for statistical analysis. The level of significance was adjusted at α ≤ 0.05.

## 3. Results

### Data Set Queries

Initially, our plan was to include a data set consisting of a first and second dental report on first, second and fourth graders. Hence, a total of 366 data sets for the control group aged five to eleven were entered into the Excel file. Of these data sets from the control group, 23 data sets contained a query regarding the dental findings. In the control group, all queries could be clarified based on the interim dental findings. For the test group, a total of 277 data sets of children aged five to eleven were entered into the Excel file. Out of the 277 data sets, 39 had a query regarding the dental findings. These 39 queries could not be clarified by interim findings, leading to the deletion of these data sets prior to further data analysis. The largest age group in the control and test groups were the 7- and 8-year-olds. Therefore, these two age groups were further analyzed (Table 1 and Table 2).

A total of 103 children aged 7 years (48 female, 46.6% and 55 male 53.4%) were included in the control group, and 89 (46 female, 51.7% and 43 male 48.3%) in the test group. A total of 112 children aged 8 years (51 female, 45.5% and 61 male 54.5%) were included in the control group and 91 (51 female, 56% and 40 male 44%) in the test group. In the control group of 7-year-olds, the mean dmf-t/DMF-T value decreased by 0.13 from the first to the second examination. In the test group, this value increased by 0.02 between the first and second examinations (Table 1). In all groups (Table 1 and Table 2), a maximum of 61% of the children with caries experience were completely treated. However, there was no significant difference between the test and control groups in either the percentage of sealed first permanent molars or the degree of restoration. For both age groups, no significant difference in caries increment in deciduous teeth between the test and control groups was found (Table 3).

Despite the interruption of group prevention with fluoride varnish application, there was no significant increase in the progression of initial lesions towards filling and decay in the deciduous and permanent dentition between test and control groups. In the total control group of 7-/8-year-olds, twenty-eight deciduous teeth showed an initial lesion in the first examination, and six (21.43%) of these lesions progressed during the observation period (three filled and three decayed). In the total test group of 7-/8-year-olds, thirty-five primary teeth showed an initial lesion, and eight (22.86%) of these lesions progressed to fillings (six) and enamel breakdown (two) in the second examination. In the control group, one (10%) out of ten initial lesion in the permanent dentition progressed to an enamel break down. In the total test group of 7-/8-year-olds, two initial lesions (16.67%) out of twelve developed into a cavity with enamel breakdown. A total of 85 initial lesions were detected in the first examination, of which 25 were remineralized in the second examination.

## 4. Discussion

In the present retrospective anonymized data analysis, no significant increase in caries in deciduous or permanent teeth was found during the COVID-19 pandemic compared to a period before the pandemic. Therefore, in the population studied, the interruption of SIP due to the COVID-19 pandemic had no negative impact on caries increment. Against this background, the null hypothesis can be accepted for the small group of schoolchildren analyzed here. However, the chosen study design of a retrospective study [18,19,20,21,22] and the sample size are the greatest limitations of the study. It is difficult to retrospectively generate an observation period that is as similar as possible for a control and a test group. This was not achieved in the present study, as shown by the significant difference in the observation period between the control and test group. Due to the abrupt interruption of group prevention, it was also no longer possible to extend the observation period for the control group. After the restart of group prevention, all those people working in the dental public health service wanted to know whether there had been any changes in oral health, so the epidemiological examinations were quickly resumed without any thought of analyzing the caries increment. Normally, the children’s caries experience, but not the caries increment, is documented as part of the epidemiological examinations. Based on the evaluation of the mean dmf-t/DMF-T values for the 7-year-olds in the first examination and for the 9-year-olds in the second examination, it becomes clear why this method is unsuitable as the single evaluation for identifying possible changes in oral health over a short observation period of two up to two and a half years. In the present analysis, there was a significant difference in this respect as the test group had a lower mean dmf-t/DMF-T value compared to the control group. This could have been caused by a small number of children with high caries experience. It is therefore recommended to carry out a dichotomized caries experience evaluation. In the present dichotomized evaluation, the large fluctuations between the percentage of children with and without caries experience are remarkable. In the examined groups, a decrease in the percentage of children with naturally sound dentitions can be found, which can be explained by the fact that the longer a tooth is in the oral cavity, the higher the probability of developing caries will be. On the other hand, the schoolchildren examined were also in the phase of mixed dentition, which can cause an increase in the percentage of children with naturally healthy dentitions. Therefore, there are natural fluctuations in the examined age group that must be considered as a bias. To counteract a distortion, an evaluation should be made based on the individual child as an assessment of the caries increment and ideally even on the individual tooth. The latter is time-consuming and is not feasible with the large amounts of data that are collected in the representative nationwide epidemiological studies.

Only one data collection is required for a measurement of caries experience, while two data collections are required to calculate the caries increment. During the restart of group prevention, many children with mild colds stayed at home, whereas before the pandemic, these children would also have gone to school. This has certainly contributed to a reduced number of data sets for the test group consisting of two individual dental evaluations. The control group exhibited a further examination between the first and the last examination. For the control group, the reports of the further examination were used to clarify queries. This was not possible in the test group. As a result, 39 data sets with queries failed the plausibility check and were removed before the statistical analysis, which in turn reduced the number of samples. The two points staying at home with a mild cold and not providing additional dental records to clarify findings queries bring with them the risk of a selection bias for the test group.

The data for this study were obtained from the records of the dental public health service. Therefore, the data extraction depended on both the amount and quality of information kept in dental records [19]. As far as the quality is concerned, only one experienced dentist (TH) performed the dental examinations and the data recording aiming at maximum homogeneity [17]. Moreover, that dentist participated in all previous calibration for the studies of the German Association for Youth Dentistry. The two last mentioned points make the evaluated data sets valuable for the comparison of oral health before the COVID-19 pandemic and during the pandemic.

For time and organizational reasons, caries was recorded based on the WHO criteria [14], whereby initial lesions were also recorded, but without separate drying with cotton rolls and compressed air. The children were asked to swallow their saliva, but in the end an initial lesion according to ICDAS code 2 was recorded, while ICDAS 1 was not considered, as no compressed air was available. Especially when it comes to treating initial lesions at an early stage with caries prevention through targeted motivation, tooth brushing training, oral health education and fluoride varnish application, it would have been better if ICDAS 1 and 2 had been accurately recorded. However, using ICDAS for examining the children’s teeth would be more time-consuming [23]. For the underlying data collection, an easily transportable device with fiber-optic transillumination could have been helpful in caries diagnosis in the permanent dentition. The authors of a Cochrane review with meta-analysis concluded that this technique has limitations in detecting enamel caries, while the identification of healthy teeth is considered successful [24]. Because of the cervical position of the contact point on the primary molars, fiber-optic transillumination is not of importance in the proximal caries detection in deciduous molars. However, bitewing radiographs are not used in the setting of a dental screening examination in schools.

The application of fluoride varnish as part of group prevention in the Marburg-Biedenkopf district is well established with parental approval of fluoride varnish application at over 90%. In socially deprived areas, children in some kindergartens also receive intensive group prevention for children [23]. Participation in this program, starting at kindergarten age, can certainly contribute to making daily tooth brushing a ritual. Only if these rituals are truly well established, there is a chance that rituals will be maintained in times of crisis such as the COVID-19 pandemic. It is speculation whether daily dental care was truly ritualized in the test group. To determine this, a near-real-time survey of the children would have been necessary. However, this was not done in the present case. In Portugal and Spain, a survey was conducted on dental care habits of 3- to 17-year-olds during the lockdown, and here most caregivers report that children’s oral hygiene habits showed no significant changes [25]. Even if the toothbrush has not adequately reached all tooth surfaces, at least fluoride has entered the child’s mouth when the teeth are cleaned with fluoride toothpaste, which contributes to remineralization [26]. This may also explain why, among all the children examined in this study, nearly 30% of the initial lesions recorded in the first findings were recorded as healthy in the second findings and were apparently remineralized.

In addition to tooth brushing behavior and fluoride intake, eating behavior is also an important factor for caries progression. It is possible that the children in this study had less access to cariogenic food during the COVID-19 pandemic than before the pandemic. It is also possible that children consumed more sugar, especially during the lockdown, to compensate for the emotional state of stress caused by online teaching and a lack of real social contact through emotional eating [27]. Since the eating habits of the children in the study were not assessed using a questionnaire, this would be pure speculation. However, studies can be found in the literature that assume a shift in eating behavior during the COVID-19 pandemic. In their survey, Costa et al. found that children with untreated dental caries consumed more snacks during the lockdown [25]. In Italy, Docimo et al. found an increase in the consumption of sweets during the lockdown, but like in Spain and Portugal, there was no significant change in toothbrushing behavior [25,28]. González-Monroy et al. investigated eating behavior during the COVID-19 pandemic in their systematic review and found a shift in eating behavior towards an increased frequency of snacking and a preference for sweets and ultra-processed foods instead of fruits, vegetables, and fresh foods [29]. The COVID-19 pandemic has affected children’s lifestyles and eating habits, leading to an increase in the prevalence of obesity, due to the intake of sugary foods and drinks, as well as a decrease in physical activity during the lockdown period [30]. However, weight gain does not necessarily go hand in hand with an increased risk of caries if toothbrushing behaviors with fluoride toothpaste lead still to sufficient tooth remineralization.

The proportion of children who had not received full oral rehabilitation was between 40% and 60%. While caries prevention is better than a filling, children with caries experience must also receive adequate treatment. There is still a need for action here. Scherrer et al. used a simulation model to examine the impact of the COVID-19 pandemic on the oral health of children from low-income households in the United States and concluded that the negative effects of the pandemic could be partially reduced by an increased access to sealants [31]. During the COVID-19 pandemic, school-based dental sealing programs in the US were interrupted. In Germany, group prevention was temporarily discontinued, but it was still possible to have individual prophylactic measures carried out in private practices. For the present analysis, it is encouraging to note that there was no significant drop in the degree of oral rehabilitation among the children examined during the pandemic, which shows that the general practitioners in the dental praxis were visited for the purpose of individual prevention.

## 5. Conclusions

It is possible that the children examined went through the pandemic without a significant increase in tooth decay because SIP includes tooth brushing training several times a year in addition to fluoridation. Although the children in the test group had no training as part of group prevention during the observation period, these children had generally already taken part in group prevention in kindergarten and had therefore already experienced 4 to 5 years of prevention and at least one year of this as an SIP.

Being trained in the selective preventive program was a valuable component in getting through the COVID-19 pandemic as dental-healthy as possible. How valuable this has actually been will depend on a comparison with other studies, which will then hopefully evaluate the caries increment on an individual child basis in addition to the easily recorded caries experience.

## Figures and Tables

**Table 1:** Results of the control and test group of the 7-year-olds at first and 9-year-olds at second dental examination regarding caries experience, erupted and sealed first permanent molars and level of oral rehabilitation.

	First Dental Examination, 7-Year-Olds			Second Dental Examination, 9-Year-Olds		
	Control Group N = 103	Test Group N = 89	*p* Value	Control Group N = 103	Test Group N = 89	*p* Value
**Mean age of** **children**	7.37 ± 0.30	7.40 ± 0.29	0.4758 *	9.37 ± 0.34	9.77 ± 0.40	<0.0001 *
**Mean dmf-t/DMF-T**	2.55 ± 3.15 minimum 0 maximum 12	1.17 ± 1.97 minimum 0 maximum 9	0.0019 *	2.42 ± 2.76 minimum 0 maximum 9	1.19 ± 2.03 minimum 0 maximum 9	
						0.0005 *
**Number of erupted first** **permanent molars**	367	340		404	356	
**Number of first** **permanent molars with** **fissure sealants**	45 (12.26%)	52 (15.29%)		112 (27.72%)	112 (31.46%)	
**Number of children with dmf-t/DMF-T = 0**	45 (43.69%)	56 (62.92%)	0.008 **	39 (37.86%)	54 (60.67%)	0.002 **
**Number of children with dmf-t/DMF-T > 0**	58 (56.31%)	33 (37.08%)		64 (62.14%)	35 (39.33%)	
**Number of children** **with 100% level of** **dental rehabilitation**	22 (37.93% of children with caries experience 21.36% of these control group)	18 (54.55% of children with caries experience 20.22% of these control group)	0.125 **	32 (50% of children with caries experience 31.07% of these control group)	21 (60% of children with caries experience 23.60% of these control group)	0.34 **
**Number of children** **with less than 100% level** **of dental rehabilitation**	36 (62.07% of children with caries experience 34.36% of these control group)	15 (45.45% of children with caries experience 16.85% of these control group)		32 (50% of children with caries experience 31.07% of these control group)	14 (40% of children with caries experience 15.73% of these control group)	
**Number of children** **with 0% level of** **dental rehabilitation**	17 (29.31% of children with caries experience 16.50% of these control group)	5 (15.15% of children with caries experience 5.62% of these control group)		14 (21.88% of children with caries experience 13.60% of these control group)	6 (17.14% of children with caries experience 6.74% of these control group)	
		

* Mann–Whitney U Test, ** Chi square Test.

**Table 2:** Results of the control and test groups for the 8-year-olds at first and 10/11-year-olds at second dental examination regarding caries experience, erupted and sealed first permanent molars and level of oral rehabilitation.

	First Dental Examination, 8-Year-Olds			Second Dental Examination, 10/11-Year-Olds		
	Control Group N = 112	Test Group N = 91	*p* Value	Control Group N = 112	Test Group N = 91	*p* Value
**Mean age of** **children**	8.97 ± 0.63	8.75 ± 0.59	0.0267 *	10.99 ± 0.70	11.00 ± 0.68	0.6799 *
**Mean dmf-t/DMF-T**	2.25 ± 2.74 minimum 0 maximum 10	2.65 ± 3.07 minimum 0 maximum 11	0.5383 *	1.49 ± 2.16 minimum 0 maximum 7	1.13 ± 1.96 minimum 0 maximum 9	
						0.2404 *
**Number of erupted first** **permanent molars**	448	358		447	364	
**Number of first** **permanent molars with** **fissure sealants**	109 (24.33%)	90 (25.14%)		176 (39.37%)	126 (34.62%)	
**Number of children with dmf-t/DMF-T = 0**	45 (40.18%)	37 (40.66%)	0.945 **	60 (53.57%)	55 (60.44%)	0.326 **
**Number of children with dmf-t/DMF-T > 0**	67 (59.82%)	54 (59.34%)		52 (46.43%)	36 (39.56%)	
**Number of children** **with 100% level of** **dental rehabilitation**	36 (53.73% of children with caries experience 32.14% of these control group)	22 (40.74% of children with caries experience 24.18% of these control group)	0.155 **	27 (51.92% of children with caries experience 24.11% of these control group)	22 (61.11% of children with caries experience 24.18% of these control group)	0.394 **
**Number of children** **with less than 100% level** **of dental rehabilitation**	31 (46.27% of children with caries experience 27.68% of these control group)	32 (59.26% of children with caries experience 35.16% of these control group)		25 (48.07% of children with caries experience 22.32% of these control group)	14 (38.89% of children with caries experience 15.38% of these control group)	
**Number of children** **with 0% level of** **dental rehabilitation**	11 (16.42% of children with caries experience 9.82% of these control group)	13 (24.07% of children with caries experience 14.29% of these control group)		10 (19.23% of children with caries experience 8.93% of these control group)	4 (11.11% of children with caries experience 4.40% of these control group)	

* Mann–Whitney U Test, ** Chi square Test.

**Table 3:** Results of the control and test groups in both age groups regarding mean period between the first and second examination and caries increment for the first and permanent dentition.

		Seven-Year-Olds at the First Examination			Eight-Year-Olds at the First Dental Examination		
		Control Group N = 103	Test Group N = 89	*p* Value	Control Group N = 112	Test Group N = 91	*p* Value
**Mean period** **between the first and second** **examination in days**		717.97 ± 43.83	877.51 ± 79.14	<0.0001 *	737.58 ± 81.29	848.43 ± 84.86	<0.0001 *
**Number of** **children (%)** **with caries increment in the first** **dentition**	**Yes**	32 (31.07%)	23 (25.84%)	0.43 **	24 (21.43)	13 (14.29%)	0.19 **
	**No**	71 (68.93%)	66 (74.16%)		88 (78.57%)	78 (85.71%)	
**Number of** **children (%)** **with caries** **increment in** **the permanent** **dentition**	**Yes**	9 (8.74%)	1 (1.12%)	0.018 **	10 (8.93%)	11 (12.09%)	0.46 **
	**No**	94 (91.26%)	88 (98.88%)		102 (91.07%)	80 (87.91%)	

* Mann–Whitney U Test, ** Chi square Test.

## Data Availability

Data is unavailable due to ethical restrictions. Legislation in the federal state of Hesse allows anonymized data analysis by third parties, but further data transfer would require the consent of parents and children. However, no informed consent was obtained.
